# Gut bacterial community profile of frugivorous bats from Cathedral Cave in Cavinti, Laguna, Philippines

**DOI:** 10.1128/mra.00426-24

**Published:** 2024-08-20

**Authors:** Bonie B. Datul, Ronilo Jose D. Flores, Andrew D. Montecillo, Marian P. De Leon

**Affiliations:** 1 Museum of Natural History, University of the Philippines Los Baños, Los Baños, Laguna, Philippines; 2 Environmental Biology Division, Institute of Biological Sciences, University of the Philippines Los Baños, Los Baños, Laguna, Philippines; 3 Microbiology Division, Institute of Biological Sciences, University of the Philippines Los Baños, Los Baños, Laguna, Philippines; California State University San Marcos, San Marcos, California, USA

**Keywords:** bat gut microbiome, bats, frugivorous bats, bacterial community profile, cave bats

## Abstract

Here we report the 16S rRNA gene amplicon analysis of the gut microbiota of three frugivorous cave bat species from the Cathedral Cave in Cavinti, Laguna, Philippines. Among the bat species, the most abundant phyla are Proteobacteria and Firmicutes D.

## ANNOUNCEMENT

Bats inhabit diverse ecological niches worldwide, from thick rainforests to busy urban areas. Despite their ecological prominence, their gut microbiota composition remains largely unexplored. The gut plays a pivotal role in bat health, metabolism, and immune function, and unraveling the composition and dynamics of the bat gut microbiota has important implications for understanding host-microbe interactions and their roles on bat physiology and ecology (1, 2). Here, we present the gut-associated bacterial community of three frugivorous bat species, namely, *Cynopterus brachyotis* (Müller, 1838), *Ptenochirus jagori* (Peters, 1861), and *Rousettus amplexicaudatus* (E. Geoffroy, 1810) from the Cathedral Cave, Cavinti Underground River and Caves Complex, Cavinti, Laguna, Philippines (14°16′54.48″ N, 121°38′9.661″ E) through 16S rRNA metabarcoding.

The gut of three bat species: *C. brachyotis* (*n* = 4), *P. jagori* (*n* = 5), and *R. amplexicaudatus* (*n* = 3) were collected in November 2021. The gut was removed at the sampling site then transported to the laboratory in liquid nitrogen. The intestines were opened, and the mucosal lining tissue with the gut contents was scraped off (3, 4) for DNA extraction using Microbiome DNA Isolation Kit (Norgen Biotek, ON, Canada). The lysis step was modified to 10 minutes at 65°C. Libraries were prepared using the Nextera XT DNA Library Preparation Kit (Illumina, San Diego, CA) following the manufacturer’s 16S Library Preparation Workflow: the V3-V4 regions of the16S rRNA gene were amplified using 341F and 805R primers with Illumina adapters (5). Sequencing was performed on Illumina MiSeq (2 × 300 bp) (5).

The demultiplexed sequence data were processed using QIIME2 (v. 2023.9) (6). Default parameters were used unless otherwise stated. DADA2 (v. 2023.9.0) (7) was used to trim the reads, resolve amplicon sequence variants (ASVs), and merge overlapping sequences. Reads were truncated based on the values obtained from FIGARO (8): p-trunc-len-f and p-trunc-len-r for *C. brachyotis*, *P. jagori*, and *R. amplexicaudatus* are 284 and 239, 289 and 234, and 296 and 227, respectively; p-max-ee-f and p-max-ee-r for all samples are both 2. Forward and reverse reads were trimmed at positions 17 and 21, respectively. Taxonomy was assigned using the multinomial naive Bayes classifier method (9) in the q2-feature-classifier plugin (v. 2023.9.0) (10) using the V3-V4 region from the Greengenes2 (v. 2022.10) reference database (11, 12). Non-bacterial ASVs were removed using the q2-taxa (6). Resulting ASVs were combined per bat species with the merge function in qiime2 (6) and used for downstream analyses in R (v.4.3.3) (13) using the phyloseq (14) and ggplot2 (15) packages.

**Fig 1:**
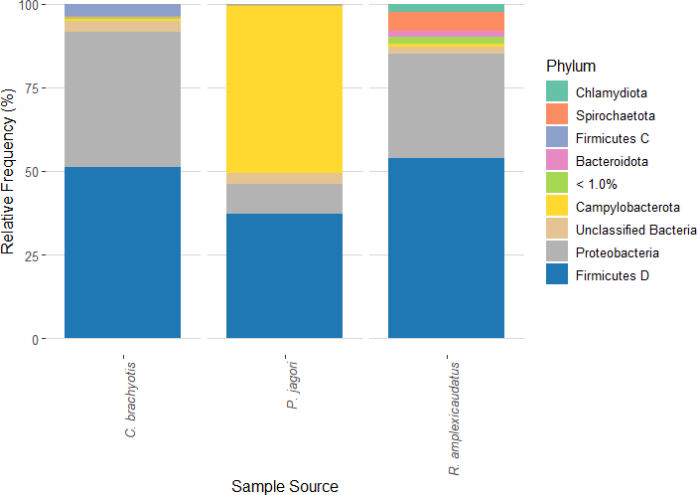
Taxa bar plots showing representative phyla composing the gut-associated bacteria of the three fruit bat species collected from Cavinti Underground River and Caves Complex determined by amplicon sequencing of the V3-V4 region of the 16S rRNA. Each bar represents the combined data from specific bat species, and each colored stacked box represents a bacterial taxon.

A combined total of 462,435, 559,663, and 378,999 paired-end reads were obtained from *C. brachyotis*, *P. jagori*, and *R. amplexicaudatus*, respectively (Table 1). The three most abundant phyla are Firmicutes D (51.01%), Proteobacteria (40.47%), and Firmicutes C (3.52%) for *C. brachyotis*, Campylobacterota (50.08%), Firmicutes D (37.07%), and Proteobacteria (8.99%) for *P. jagori*, and Firmicutes D (53.74%), Proteobacteria (31.32%), and Spirochaetota (5.38%) for *R. amplexicaudatus* (Fig. 1). It is noteworthy that the gut bacterial community diversity among the bat species was different.

**TABLE 1:** Sequence Read Archive accession number and library statistics through filtering, denoising, merging, and chimeric and non-bacterial sequence removal of V3-V4 region amplicons from frugivorous cave bats

Library name	Host bat species	Input	Filtered	Denoised	Merged	Input merged (%)	Non- chimeric	Percentage of bacterial sequences (%)	SRA accession no.
Cbra1001	*C. brachyotis*	109,480	91,267	91,005	90,085	82.28	75,820	69.25	SRR28556661
Cbra1002	*C. brachyotis*	119,356	102,065	101,940	101,877	85.36	101,525	85.06	SRR28556660
Cbra1003	*C. brachyotis*	103,575	86,831	86,665	86,265	83.29	82,051	79.22	SRR28556649
Cbra1004	*C. brachyotis*	130,024	110,188	110,031	109,589	84.28	106,043	81.56	SRR28556638
Pjag1001	*P. jagori*	107,007	86,977	86,630	86,425	80.77	84,759	79.21	SRR28556627
Pjag1002	*P. jagori*	60,987	45,914	45,488	44,751	73.38	43,271	70.95	SRR28556624
Pjag1003	*P. jagori*	164,588	142,256	142,195	142,137	86.36	141,670	86.08	SRR28556623
Pjag1004	*P. jagori*	119,773	103,600	103,313	102,229	85.35	97,581	81.47	SRR28556622
Pjag1005	*P. jagori*	107,308	91,385	91,329	91,154	84.95	90,336	84.18	SRR28556621
Ramp1001	*R. amplexicaudatus*	131,413	107,852	107,593	106,988	81.41	101,948	77.58	SRR28556620
Ramp1002	*R. amplexicaudatus*	117,861	97,092	96,924	96,089	81.53	92,275	78.29	SRR28556659
Ramp1003	*R. amplexicaudatus*	129,725	107,828	107,726	107,628	82.97	107,057	82.53	SRR28556658

## Data Availability

The samples were uploaded to the National Center for Biotechnology Information under BioProject accession number PRJNA1096155. The accession numbers for all 12 SRA files are as follows: SRR28556620, SRR28556621, SRR28556622, SRR28556623, SRR28556624, SRR28556627, SRR28556638, SRR28556649, SRR28556658, SRR28556659, SRR28556660, and SRR28556661.
